# Antineutrophil cytoplasmic antibody positivity and clinical implications in COVID-19

**DOI:** 10.2217/fvl-2021-0125

**Published:** 2022-02-10

**Authors:** Serdar Can Güven, Hakan Apaydın, Bahar Özdemir, Berkan Armağan, İhsan Ateş, Abdulsamet Erden, Orhan Küçükşahin, Ahmet Omma

**Affiliations:** ^1^Ministry of Health Ankara City Hospital, Clinic of Rheumatology, Ankara, 06800, Turkey; ^2^University of Health Sciences, Ankara City Hospital, Department of Internal Medicine, Ankara, 06800, Turkey; ^3^Yıldırım Beyazıt University Medical School, Department of Internal Medicine, Division of Rheumatology, Ankara, 06800, Turkey; ^4^University of Health Sciences, Ankara City Hospital, Clinic of Rheumatology, Ankara, 06800, Turkey

**Keywords:** ANCA, antineutrophil cytoplasmic antibody, COVID-19, mortality, outcome

## Abstract

**Aim:** To investigate clinical implications of antineutrophil cytoplasmic antibody (ANCA) positivity detected in COVID-19 patients during follow up. **Materials and methods:** A retrospective survey in a hospital database was carried out to detect COVID-19 patients in which ANCAs had been tested. Clinical, laboratory and imaging data were collected from this hospital database and compared between ANCA-negative and -positive patients. **Results:** ANCAs were tested in 87 COVID-19 patients. Eight had positivity in at least one ANCA test. COVID-19 symptoms on admission and rate of pulmonary involvement were similar. Acute phase reactant levels were higher in ANCA-positive patients. Rate of mortality was higher in the ANCA-positive group without statistical significance. **Conclusion:** ANCA positivity detected during COVID-19 in patients without a prior diagnosis of any rheumatic condition may be related with worse outcomes.

The SARS-CoV-2 pandemic caused a global health crisis after the first human cases reported in December 2019. COVID-19 has a heterogeneous course and despite the fact that most patients suffer mildly, a considerable number of cases develop mortal complications such as pneumonia, acute respiratory distress syndrome, thrombosis and a hyperinflammatory state called cytokine storm, which is basically an exaggerated response of innate immunity triggered by viral proteins [1–3]. During the natural course of viral infections, the initial innate response to pathogen-associated molecular patterns (in this case membrane proteins of SARS-CoV 2) is followed by recognition of viral particles by humoral immune system after a cascade of signaling activity and activation of T and B lymphocytes [4]. This process results in antigen-specific neutralizing antibody production which is crucial for viral clearance. However, similar to other viral pathogens like EBV and parvovirus, SARS-CoV-2 reportedly alter the ability of humoral immune system to distinguish self antigens and trigger the production of pathogenic autoantibodies like antinuclear and antiphospholipid antibodies [5].

Antineutrophil cytoplasmic antibodies (ANCAs) are autoantibodies directed against various neutrophil antigens such as lactoferrin, cathepsin, elastase, myeloperoxidase (MPO) and proteinase 3 (PR3). Among these, particularly ANCAs against MPO and PR3 are related with development of ANCA-associated vasculitis (AAV). The exact mechanisms which trigger the formation of pathogenic ANCAs are yet to be clarified. Infectious diseases are suggested as one of the major causes via mechanisms like stimulating neutrophil extracellular trap (NET) formation, molecular mimicry and breaking self-tolerance to autoantigens as an end result [6,7]. Epitope sharing, spreading and genetic susceptibility (*HLA* subtypes and polymorphisms in *SERPINA1*, *PR3*, *CTLA4*, *PTPN22* genes) are also thought to play a role in altered self-tolerance [6,8,9]. When neutrophils are activated, for instance in presence of an acute infection, expression of membrane-bound MPO and PR3 is enhanced, which are more accessible for circulating MPO and PR3 ANCAs to bind than cytosolic MPO and PR3 [10,11]. This interaction results in abnormal neutrophil activity and enhanced neutrophil–endotel interaction leading to microvascular damage [10,12].

 SARS-CoV-2 infection had been demonstrated to be related with *de novo* autoantibody production possibly via causing immune dysregulation [5,13]. However, there is scarce data regarding frequency of new onset ANCA positivity in COVID-19 or possible clinical implications of *de novo* ANCA production [5,13,14]. Here in this study, we aimed to investigate presence of ANCA positivity in COVID-19 patients and possible relations with clinical findings and COVID-19 outcomes.

## Materials & methods

We carried out a survey in our hospital database as of January 2021, to detect COVID-19 patients in which ANCAs had been tested. We screened for patients in which International Classification of Disease codes for COVID-19 (U06.0 and U07.3) and laboratory codes for ANCA tests (immunofluorescent assay [IFA] or PR3/MPO enzyme-linked immunosorbent assay [ELISA] [906.770, 907.950, 907.840]) were simultaneously present. Among these, patients who had a positive SARS-CoV-2 PCR test result, in which any ANCA testing (IFA or PR3-ELISA or MPO-ELISA) was executed within 3 months after positive PCR result, were enrolled. Patients younger than 18 years of age, who were pregnant during COVID-19 infection, patients with a diagnosis of any rheumatic condition, autoimmune disease or malignancy prior to COVID-19 and patients who had been tested for ANCAs before SARS-CoV-2 PCR test were excluded.

In IFA perinuclear (pANCA) or cytoplasmic (cANCA) staining in titres ≥ 1:10 dilution was accepted as positive and the cut-off value for PR3 and MPO ELISA positivity was ≥ 19.99 RU/ml according to our laboratory reference values.

Demographics and comorbidities, COVID-19 symptoms on admission, laboratory findings, thorax computed tomography (CT) results and COVID-19 outcomes were investigated via thehospital database and recorded for each patient. For each laboratory parameter, the worst value during follow up was recorded. Data were compared between ANCA-negative and -positive patients.

Statistical analyses were made using Statistical Package for the Social Sciences v22. Continuous variables were presented with median (interquartile range [IQR]) values and compared between groups by Mann–Whitney U test. Categorical variables were expressed with numbers (percentages) and compared between groups by chi-squared test. p-values ≤ 0.05 were accepted as statistically significant.

## Results

A total of 87 patients, who underwent ANCA testing (either IFA or ELISA or both) during COVID-19 follow up, were enrolled. Eight of them had positivity in at least one ANCA test (eight in IFA with two pANCA and six cANCA staining, one additionally in ELISA with positivity for PR3 matching with 1:320 titer cANCA staining in IFA) (Supplementary Table 1). Seven patients had positive IFA alone, ELISA tests had been screened in three patients with a single positivity for PR3. None of the enrolled patients had a known diagnosis of any rheumatic condition prior to COVID-19 infection. ANCA positivity was detected during hospital stay for COVID-19 in all eight ANCA-positive patients.

In all patients, the most common reasons for ANCA testing were elevated serum creatinin levels (58.6%), cavitary lesions in thorax CT (5.7%) and persistent fever/elevated acute phase reactants (5.7%). In the ANCA-positive group, most common reason was again elevated serum creatinine levels (75.0%) (Supplementary Table 1).

Demographics, smoking status, comorbidities of ANCA-negative and -positive patients were presented in Table 1. The ANCA-positive group was inclined to have older age and increased rate of male patients, yet neither reached statistical significance. The number of patients with at least one comorbidity and at least two comorbidities were insignificantly higher in the ANCA-negative group. Diabetes mellitus was more frequent in the ANCA-negative group (36.7% vs 0%; p = 0.036).

**Table 1.** Demographics, comorbidities, COVID-19 symptoms, thorax computed tomography findings in patients with and without ANCA positivity.

	ANCA negative (n = 79)	ANCA positive (n = 8)	p-value
Age, years, median (IQR)	66.0 (20.0)	74.5 (40.5)	0.328
Gender, male, n (%)	42 (53.2)	6 (75.0)	0.237
Patients with ≥1 comorbidities, n (%)	69 (87.3)	6 (75.0)	0.335
Patients with ≥2 comorbidities, n (%)	45 (56.9)	2 (25.0)	0.084
**Comorbidities, n (%)**			
Hypertension	52 (65.8)	3 (37.5)	0.113
Diabetes	29 (36.7)	0 (0)	**0.036**
Asthma/chronic obstructive pulmonary disease	14 (17.7)	1 (12.5)	0.709
Chronic kidney disease	16 (20.3)	1 (12.5)	0.598
Coronary artery disease	13 (16.5)	2 (25.0)	0.542
Congestive heart failure	9 (11.4)	0 (0)	0.313
Cerebrovascular event	3 (3.8)	1 (12.5)	0.263
Active smokers, number (%)^†^	6 (13.6)	1 (12.5)	0.698
**Symptoms on admission, n (%)**			
Fever	29 (36.7)	4 (50.0)	0.460
Malaise	62 (78.5)	6 (75.0)	0.820
Cough	53 (67.1)	4 (50.0)	0.333
Dyspnea	48 (60.8)	3 (37.5)	0.203
Hemoptysis	4 (5.1)	1 (12.5)	0.389
Myalgia	35 (44.3)	6 (75.0)	0.097
Arthralgia	16 (20.3)	1 (12.5)	0.598
Abdominal pain	12 (15.2)	2 (25.0)	0.472
Diarrhea	8 (10.1)	1 (12.5)	0.834
Headache	10 (12.7)	0 (0)	0.285
Anosmia	9 (11.4)	0 (0)	0.313
Ageusia	11 (13.9)	0 (0)	0.259
Pulmonary involvement in thorax computed tomography, n (%)^‡^	72 (92.3)	7 (87.5)	0.636
**Thorax computed tomography findings, n (%)^‡^**			
Multilober involvement	67 (85.9)	7 (87.5)	0.697
Ground-glass opacity	49 (62.8)	4 (50.0)	0.478
Cavity	5 (6.4)	0 (0.0)	0.461
Consolidation	16 (20.5)	2 (25.0)	0.766
Crazy paving	14 (17.9)	0 (0)	0.190
Three-in-bud	2 (2.6)	0 (0)	0.647
Septal thickening	2 (2.6)	1 (12.5)	0.145
Hemorrhage	1 (1.3)	1 (12.5)	**0.045**
Honeycombing	2 (2.6)	0 (0)	0.647

Bold Values indicate statistical significance.

† Evaluated over 44 patients in the ANCA-negative group and over four patients in the ANCA-positive group due to missing data.

‡ Evaluated in 86 patients, since in one patient from the ANCA-negative group computed tomography result could not be reached.

ANCA: Antineutrophil cytoplasmic antibody; IQR: Interquartile range.

Distribution of COVID-19 symptoms on admission was similar between groups (Table 1). The most common symptoms were malaise, cough, dyspnea, myalgia and fever in both groups. Results of thorax CT examinations could be obtained for all patients except for a single patient with negative ANCAs. When a total of 86 thorax CT reports were examined, the number of patients with pulmonary involvement was similar between groups (92.3% vs 87.5%; p = 0.721) (Table 1). Majority of patients had multilobar involvement (87.5% vs 85.9%; p = 0.697). Most common types of pulmonary lesions were ground glass opacities (62.8% vs 50%; p = 0.478) and consolidations (25% vs 20.5%; p = 0.766) in both groups. None of the ANCA-positive patients had cavitary lesions or crazy-paving pattern. Alveolar hemorrhage was more frequent in ANCA-positive patients (12.5% vs 1.3%; p = 0.045) (Table 1).

Laboratory parameters were presented in Table 2. Renal functional tests were observed to be impaired in both groups without significant difference. Median C-reactive protein (CRP) and erythrocyte sedimentation rate levels were significantly increased in ANCA-positive patients while hemoglobin levels were insignificantly reduced. Moreover, presence of hematuria in urinalysis was more frequent in the ANCA-positive group (75.0% vs 43.0%; p = 0.084). Procalcitonin levels were also observed to be higher in the ANCA-positive group despite not reaching statistical significance (median [IQR], 3.7 [15.7] vs 0.58 [6.34]; p = 0.269); however, number of patients with a positive bacterial culture from any body fluid (including blood, urine, sputum and bronchial aspirate etc.) was similar between groups (54.4% vs 62.5%; p = 0.662). None of the patients in the entire population had eosinophilia. Additionally, in seven of the eight ANCA-positive patients we observed that IFA for presence of antinuclear antibodies (ANA) had also been screened with low titer positivity in two (both with a titer of 1:100, one with granular staining and the other with homogenous staining, neither of them had been screened for presence of extractable nuclear antibodies).

**Table 2.** Laboratory findings in patients with and without antineutrophil cytoplasmic antibody positivity.

	ANCA negative (n = 79)	ANCA positive (n = 8)	p-value
Hemoglobin, g/dl, median (IQR)	10.4 (3.7)	8.6 (4.2)	0.061
White blood count, per mm^3^, median (IQR)	8000 (8025)	7920 (5845)	0.201
Lymphocytes, per mm^3^, median (IQR)	640 (815)	655 (462.5)	0.895
Neutrophils, per mm^3^, median (IQR)	6100 (6845)	6535 (5690)	0.228
Eosinophils, per mm^3^, median (IQR)	50 (110)	55 (242.5)	0.653
Platelets, per mm^3^, median (IQR)	234000 (130000)	165500 (58750)	0.366
Erythrocyte sedimentation rate (mm/h), median (IQR)	50 (64.5)	83 (43)	**0.022**
C-reactive protein (mg/l), median (IQR)	150 (121.5)	232.5 (113.5)	**0.004**
IL-6 (pg/ml), median (IQR)	66 (159.5)	349 (2396.3)	0.445
Fibrinogen (g/l), median (IQR)	4.8 (2.7)	2.9 (3.9)	0.231
Ferritin (μg/l), median (IQR)	793 (1031)	1918 (1365.8)	0.071
D-Dimer (mg/l), median (IQR)	2.15 (5.95)	2.8 (22.9)	0.577
Troponin I (ng/l), median (IQR)	42 (160)	56.5 (6307.5)	0.699
NT-proBNP (ng/l), median (IQR)	618 (4553)	937.5 (14745)	0.755
Procalcitonin (μg/l), median (IQR)	0.58 (6.34)	3.7 (15.7)	0.269
Alanine aminotransferase (u/l), median (IQR)	46 (55)	43 (1092.3)	0.831
Aspartate aminotransferase (u/l), median (IQR)	50 (54)	106.5 (1069.3)	0.971
Creatinine (mg/dl), median (IQR)	1.8 (8.8)	2.75 (2.3)	0.246
Glomerular filtration rate ml/min, median (IQR)	29 (56)	19.5 (14.3)	0.394
Hematuria (erythrocyte >4/HPF in urinalysis), n (%)	34 (43.0)	6 (75.0)	0.084
Erythrocyte count in urinalysis^†^, median (IQR)	54 (236)	146 (249.3)	0.517
Positive bacterial culture from anybody-fluid during follow up, number (%)	43 (54.4)	5 (62.5)	0.662

For each parameter worst value during follow up was recorded.

Boldface values indicate statistical significance.

† Only in patients with erythrocyte > 4/HPF in urinalysis.

ANCA: Antineutrophil cytoplasmic antibody; HPF: High-power field; IQR: Interquartile range; NT-proBNP: N-terminal prohormone of brain natriuretic peptide.

All patients in our study were observed to be hospitalized. Length of hospital stay was similar between ANCA-negative and -positive groups (median [IQR] days: 20 (16.5) versus 22 (17.3); p = 0.112). Frequency of ICU admissions was higher in the ANCA-positive group without statistical significance (87.5% vs 51.9%; p = 0.054). Six out of eight patients in the ANCA-positive group were observed to be dead with a mortality rate of 75.0% which was higher in comparison to ANCA negative group (75.0% vs 43.0%; p = 0.084) (Table 3).

**Table 3.** COVID-19 outcomes in patients with and without antineutrophil cytoplasmic antibody positivity.

	ANCA negative (n = 79)	ANCA positive (n = 8)	p-value
Length of hospital stay (days), median (IQR)	20 (16.5)	22 (17.3)	0.112
Intensive care unit admission, n (%)	41 (51.9)	7 (87.5)	0.054
Length of intensive care unit stay (days), median (IQR)	12.5 (15)	9 (10.8)	0.168
Intubation, n (%)	36 (45.6)	6 (75.0)	0.112
Mortality, n (%)	34 (43.0)	6 (75.0)	0.084

ANCA: Antineutrophil cytoplasmic antibody; IQR: Interquartile range.

To further investigate factors which can potentially be related with mortality, ANCA-positive and -negative patients with mortality were compared (34 ANCA negative vs 6 ANCA positive) (Supplementary Table 2). Although the frequencies of comorbidities and bacterial culture positivity were observed to be increased in the ANCA-negative group, none of these in addition to age, gender, number of active smokers and number of patients with pulmonary involvement were significantly different from the ANCA-positive group.

Thrombotic events were detected in four patients during follow up, all of which were ANCA negative. One of the ANCA-positive patients were detected to undergo a kidney biopsy revealing immunoglobulin (Ig) A nephropathy without any histopathologic findings compatible with vasculitis. Distribution of immunosuppressive and immunomodulatory treatment agents used for COVID-19 was similar between groups (Supplementary Table 3).

## Discussion

Our results imply worse outcomes in COVID-19 patients with ANCA positivity detected during follow up, who did not have a diagnosis for any rheumatic condition prior to COVID-19. The most common reason for testing ANCAs was altered renal function. Demographics, frequency of comorbidities, COVID-19 symptoms and rates of pulmonary involvement was similar between ANCA-positive and -negative patients. Presence of alveolar hemorrhage in thorax CT tended to be more frequent in ANCA-positive patients. Similarly, acute phase reactants were higher and hematuria was more frequent in the ANCA-positive group.

Data regarding the potential triggering effect of SARS-CoV 2 on *de novo* production of ANCAs is scarce in the literature. Vlachoyiannopoulos *et al.* [13] tested the sera of 29 severe COVID-19 cases without any known rheumatic conditions, revealing IFA positivity for ANCAs in four cases (13.8%, two pANCA and two cANCA staining, none of which had positive ELISA neither for MPO nor for PR3). Sacchi *et al.* [5] investigated development of several autoantibodies in 40 COVID-19 patients and reported ANCA positivity in ten (25%) with nine of them being positive for atypical ANCAs (only one had PR3 positivity). Pascolini *et al.* [14] could not detect ANCA positivity in 33 patients. In our study, among COVID-19 patients who were tested for ANCAs within 1 month after SARS-CoV-2 PCR positivity, eight (9.2%) had at least one positive ANCA test result (eight in IFA and one additionally in ELISA).

Some bacterial and viral infections are known causes for development of ANCAs [6]. Furthermore, COVID-19 had previously been related with development of various autoantibodies such as ANAs and antiphospholipid antibodies [5]. It is a well known fact that SARS-CoV-2 can trigger a hyperinflammatory state with immune dysregulation in a substantial number of COVID-19 patients. An excessive and elongated inflammatory state can be hypothesized as a cause for alteration of self-tolerance and production of autoantibodies [6,7]. In our study, inflammatory markers (CRP and erythrocyte sedimentation rate) were significantly higher in the ANCA-positive group. These findings may be reflecting a disease course with increased inflammatory burden which may be associated with development of ANCAs. Concomitant bacterial infections may also enhance ANCA production; however, number of patients with any positive bacterial culture was similar between groups in our study.

Presence of ANAs may interfere with evaluation of ANCAs by IFA leading to false-positive results. As mentioned before, SARS-CoV-2 had been reported to induce development of ANAs, likewise. In our study seven of the eight ANCA-positive patients had also been screened for presence of ANA positivity with IFA and only two low titer positive results were detected. Therefore, risk of misinterpretation of ANCA IFA due to presence of abundant ANAs may be assumed as low in our subjects.

Possible effects of ANCA development on COVID-19 outcomes were mentioned by Sacchi *et al.* [5] who reported 40% (four out of ten) mortality rate in ANCA -positive patients. Likewise, in our study, although a high mortality rate (43%) was also observed in the ANCA-negative group, which is possibly because ANCA testing was ordered in complicated patients, rates of mortality (75%) and ICU admissions were further increased in ANCA-positive patients. Outcomes in COVID-19 can also be affected by various confounders. Yet, the frequency of some major factors for worse outcomes (age, gender, frequency of comorbid diseases, smoking status, frequency of pulmonary involvement) were similar between ANCA-positive and -negative patients with mortality in our study.

It is intriguing whether ANCA positivity is merely an indicator for a severe COVID-19 course or contributes to worse outcomes by development of clinical features compatible with AAV. Several reports imply a relation between COVID-19 infection and development of new onset AAV [15–18]. Furthermore, COVID-19 and AAV share some similar features, particularly regarding pulmonary involvement, which seemingly created a diagnostic challenge for AAV during the pandemic [19–21]. In our study, presence of hematuria (43.0% vs 75.0%; p = 0.084) and alveolar hemorrhage reported in thorax CT (1.3% vs 12.5%; p = 0.045) were observed to be increased in ANCA-positive patients which may be indicative of a vasculitic process. None of our ANCA-positive patients had eosinophilia, which could have been indicative of eosinophilic granulomatosis with polyangiitis development [19]. Moreover, histopathologic evaluation did not reveal a vasculitic involvement in the ANCA-positive patient who underwent renal biopsy in our study and none of the patients, in whom alveolar hemorrhage was reported in imaging, underwent bronchoalveolar lavage for confirmation. The data we were able to gather for the study was not sufficient to retrospectively diagnose the ANCA-positive patients with AAV. Since most of the ANCA-positive patients were dead, we also could not confirm prospectively. Therefore, it remained uncertain whether ANCA positivity resulted in AAV in our patients.

There are several limitations of our study in addition to small sample size and retrospective design. First, our study is not suitable to reveal true incidence of *de novo* ANCA positivity in COVID-19. Despite the fact that we excluded patients with a prior diagnosis for any rheumatic condition, pre-COVID-19 ANCA status of the subjects are unknown. Therefore, subclinical ANCA positivity prior to infection period can not be ruled out. The majority of our patients had a clinical reason for ANCA testing, most commonly altered renal function, which creates an inevitable selection bias. Furthermore, some of our patients had been tested either for ANCA-IFA or ANCA-ELISA alone which may cause underestimation of ANCA-positive cases. Another limitation was the small sample size of the ANCA-positive group, which prevented further statistical investigations with analyses like multivariate or regression to better demonstrate effects of ANCA positivity on outcomes. Nevertheless, we investigated possible major confounders between ANCA-positive and -negative patients with mortality and did not observe any significant difference. At last, we could not gather enough data retrospectively to determine whether our ANCA-positive patients met any AAV classification criteria or not.

## Conclusion

ANCA positivity detected during COVID-19 in patients without a prior diagnosis of any rheumatic condition including AAV seemed to be related with worse outcomes. However, it is unclear whether this is due to development of vasculitic features or just a reflection of a more severe COVID-19 infection. Prospective studies with larger sample size would elucidate the true causality better.

**Summary points**

- Antineutrophil cytoplasmic antibodies (ANCAs) are autoantibodies directed against various neutrophil antigens.
- ANCAs are associated with development of ANCA-associated vasculitis.
- Infectious diseases are suggested as one of the major causes of ANCA production via mechanisms like stimulating neutrophil extracellular trap formation, molecular mimicry and breaking self-tolerance to autoantigens.
- SARS-CoV-2 infection has been demonstrated to be related with *de novo* autoantibody production.
- There is scarce data regarding *de novo* ANCA positivity in COVID-19 and clinical implications.
- In our study, among COVID-19 patients without a prior diagnosis for any rheumatic condition who were tested for ANCAs during hospital stay, ANCA positivity was detected in 9.2% of the patients.
- Inflammatory markers were significantly higher in ANCA-positive COVID-19 patients possibly reflecting an increased inflammatory burden which may be associated with development of ANCAs.
- Frequency of findings reflecting small vessel involvement such as alveolar hemorrhage and hematuria was more frequent in ANCA-positive patients.
- Mortality rate was increased ANCA-positive COVID-19 patients (75.0% vs 43.0%) without reaching statistical significance.
- It is unclear whether this is due to development of vasculitic features or just a reflection of a more severe COVID-19 infection.
- Prospective studies with larger size would elucidate the true causality better.
