# Supplementary material for: Antineutrophil cytoplasmic antibody positivity and clinical implications in COVID-19
Source: Future Virol. 2022 Feb 10:10.2217/fvl-2021-0125. doi: 10.2217/fvl-2021-0125 (PMC8833000; doi:10.2217/fvl-2021-0125)
Supplement: Supplementary file 1 [file supplementary_tables.docx]

**Supplementary Table 1. ANCA status and reasons for ANCA testing in patients**

|  | **All patients (n=87)** | |
| --- | --- | --- |
| **Any positive ANCA test, n(%)** | 8 (9.2) | |
| **Positive ANCA-IFA, n(%)** | 8 (9.2) | |
| pANCA | 2 (2.3) | |
| cANCA | 6 (6.9) | |
| **Titers of ANCA-IFA in ANCA positive patients, n(%)** |  | |
| 1:10 | 3 (37.5) | |
| 1:32 | 2 (25.0) | |
| 1:100 | 2 (25.0) | |
| 1:320 | 1 (12.5) | |
| **Positive ANCA-ELISA, n(%)*** |  | |
| MPO | 0 (0.0) | |
| PR3 | 1 (1.1) | |
| **Reasons for ANCA testing, n(%)** | **All patients**  **(n=87)** | **ANCA positive**  **(n=8)** |
| Elevated serum creatinin | 51 (58.6) | 6 (75.0) |
| Pulmonary cavity in thorax CT | 5 (5.7) | 0 (0.0) |
| Persistent fever or elevated acute phase reactants | 5 (5.7) | 1 (12.5) |
| Elevated serum creatinine with hematuria | 2 (2.3) | 0 (0.0) |
| Hemoptysis/alveolar hemorrhage in thorax CT | 2 (2.3) | 0 (0.0) |
| Pulmonary-renal syndrome | 2 (2.3) | 1 (12.5) |
| Rash | 2 (2.3) | 0 (0.0) |
| Hemolytic anemia | 2 (2.3) | 0 (0.0) |
| Elevated serum transaminases | 2 (2.3) | 0 (0.0) |
| Central nervous system lesions | 2 (2.3) | 0 (0.0) |
| Elevated serum creatinine with proteinuria | 1 (1.1) | 0 (0.0) |
| Pericarditis | 1 (1.1) | 0 (0.0) |
| Lung abscess | 1 (1.1) | 0 (0.0) |
| Honeycombing in thorax CT | 1 (1.1) | 0 (0.0) |
| Pancreatitis | 1 (1.1) | 0 (0.0) |
| Unknown | 7 (8.0) | 0 (0.0) |

**ELISA tests had been screened only in three patients, ANCA: antineutrophil cytoplasmic antibody, IFA: immunoflorescent assay, ELISA: enzyme-linked immunosorbent assay, MPO: myeloperoxidase, PR3: proteinase 3, CT: computed tomography*

**Supplementary Table 2. Comparison of factors that can be associated with mortality between ANCA negative and ANCA positive patients with mortality**

|  | **ANCA negative patients with mortality**  **(n=34)** | **ANCA positive patients with mortality**  **(n=6)** | **p** |  |
| --- | --- | --- | --- | --- |
| **Age, years, median (IQR)** | 72.5 (14.5) | 74.5 (22.3) | 0.618 |  |
| **Gender, male, number (%)** | 19 (55.9) | 4 (66.7) | 0.622 |  |
| **Patients with ≥1 comorbidities, number (%)** | 33 (97.1) | 5 (83.3) | 0.155 |  |
| **Active smokers, number (%)*** | 3 (23.1) | 1 (33.3) | 0.796 |  |
| **Pulmonary involvement in thorax computed tomography, number (%)** | 31 (91.2) | 5 (83.3) | 0.555 |  |
| **Positive bacterial culture from anybody-fluid during follow-up, number (%)** | 27 (79.4) | 4 (66.7) | 0.491 | |

**Evaluated over 13 patients in ANCA negative group and over 3 patients in ANCA positive group due to missing data, IQR: interquartile range, COVID-19: coronavirus disease 19, ANCA*: *antineutrophil cytoplasmic antibody*

**Supplementary Table 3. Distribution of treatment agents used for COVID-19 in patients**

|  | **ANCA**  **negative**  **(n=79)** | **ANCA positive**  **(n=8)** | **p** |
| --- | --- | --- | --- |
| Favipiravir, number (%) | 75 (94.9) | 8 (100) | 0.515 |
| Remdesivir, number (%) | 2 (2.5) | 0 (0) | 0.649 |
| Acetylsalicilic acid, number (%) | 27 (34.2) | 1 (12.5) | 0.211 |
| Dipyridamol, number (%) | 1 (1.3) | 1 (12.5) | 0.043 |
| Low molecular weight heparin, number (%) | 75 (94.9) | 7 (87.5) | 0.389 |
| Systemic glucocorticoids, number (%) | 51 (64.6) | 5 (62.5) | 0.908 |
| Systemic glucocorticoid pulses, number (%)* | 20 (25.3) | 4 (50.0) | 0.137 |
| Cumulative gluocorticoid dose, mg, median (IQR) | 800 (880) | 795 (2550) | 0.880 |
| Colchicine, number (%) | 16 (20.3) | 1 (12.5) | 0.598 |
| Hydroxychloroquine, number (%) | 19 (24.1) | 1 (12.5) | 0.459 |
| Anakinra, number (%) | 2 (2.5) | 1 (12.5) | 0.141 |
| Tocilizumab, number (%) | 2 (2.5) | 0 (0) | 0.649 |
| Intravenous immunoglobulin, number (%) | 2 (2.5) | 1 (12.5) | 0.141 |
| Convalescent plasma, number (%) | 1 (1.3) | 0 (0) | 0.749 |

**Pulse methylprednisolone administration with a dose ≥ 250 mg, ANCA: antineutrophil cytoplasmic antibody, IQR: interquartile range*
